# Talking with Your (Artificial) Hands: Communicative Hand Gestures as an Implicit Measure of Embodiment

**DOI:** 10.1016/j.isci.2020.101650

**Published:** 2020-10-06

**Authors:** Roni O. Maimon-Mor, Emeka Obasi, Jenny Lu, Nour Odeh, Stephen Kirker, Mairéad MacSweeney, Susan Goldin-Meadow, Tamar R. Makin

**Affiliations:** 1 Institute of Cognitive Neuroscience, University College London, 17 Queen Square, London WC1N 3AZ, UK; 2 WIN Centre, Nuffield Department of Clinical Neuroscience, University of Oxford, Oxford OX3 9DU, UK; 3 Department of Psychology, University of Chicago, Chicago, IL 60637, USA; 4 Addenbrooke's Rehabilitation Clinic, Cambridge University Hospitals NHS Trust, Cambridge CB2 0DA, UK; 5 Deafness, Cognition and Language Research Centre, University College London, London WC1H 0PD, UK

**Keywords:** Human-Centered Computing, Social Sciences, Research Methodology Social Sciences

## Abstract

When people talk, they move their hands to enhance meaning. Using accelerometry, we measured whether people spontaneously use their artificial limbs (prostheses) to gesture, and whether this behavior relates to everyday prosthesis use and perceived embodiment. Perhaps surprisingly, one- and two-handed participants did not differ in the number of gestures they produced in gesture-facilitating tasks. However, they did differ in their gesture profile. One-handers performed more, and bigger, gesture movements with their intact hand relative to their prosthesis. Importantly, one-handers who gestured more similarly to their two-handed counterparts also used their prosthesis more in everyday life. Although collectively one-handers only marginally agreed that their prosthesis feels like a body part, one-handers who reported they embody their prosthesis also showed greater prosthesis use for communication and daily function. Our findings provide the first empirical link between everyday prosthesis use habits and perceived embodiment and a novel means for implicitly indexing embodiment.

## Highlights

• Daily prosthesis use associated with greater perceived prosthesis embodiment
• Communicative gestures were applied as an implicit measure of embodiment
• Individuals missing a hand gesture more with their intact hand relative to controls
• Prosthesis gesture behavior relates to its daily functional use and embodiment

## Graphical Abstract

**Figure:**
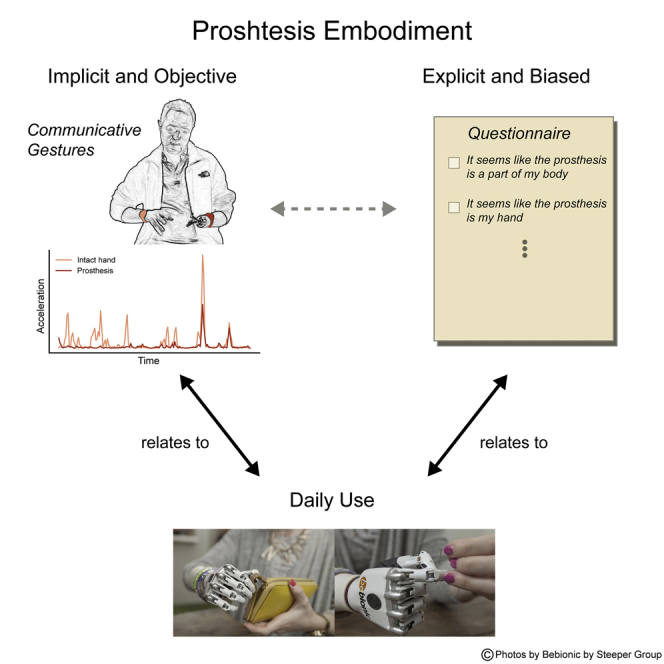

## Introduction

The notion of embodiment—which we can relate to an external and foreign object as if it was a part of our body—is increasingly capturing the interest of researchers across multiple fields. Psychologists and philosophers attempt to define and characterize embodiment (de Vignemont, 2018, 2011; Ehrsson, 2020; Longo et al., 2008; Miller et al., 2018), cognitive neuroscientists are searching for its neural fingerprint (Collins et al., 2017; Maimon-Mor and Makin, 2020; Van Den Heiligenberg et al., 2018), and biomedical and robotics engineers are interested in harnessing embodiment as a tool to measure technology adoption and successful rehabilitation (Bensmaia and Miller, 2014; Marasco et al., 2018; Pazzaglia and Molinari, 2016; Valle et al., 2018). However, despite this growing interest, the underlying mechanisms of embodiment—sharing neurocognitive resources, originally devoted to controlling one's body, to represent and operate external objects—are still poorly understood.

Perhaps the most likely candidates to achieve embodiment are substitution devices, such as artificial limbs (van den Heiligenberg et al., 2017). Artificial limb technologies attempt to increasingly mirror the appearance and function of the human body (Vujaklija et al., 2016), with hopes that greater similarity between the artificial and natural limbs will enable users to achieve “technological embodiment” (Makin et al., 2017). Embodiment has been suggested to promote intuitive control, learning, and comfort when using new tools, thus providing unique opportunities to improve the user interface for devices such as artificial limbs (Makin et al., 2017). However, there is currently little empirical evidence to show that embodiment actually relates to everyday behavior with artificial limbs, let alone that embodiment benefits users (Bekrater-Bodmann, 2020). A first challenge with filling in this empirical gap is that embodiment is a compound phenomenon, involving features that are both explicit (e.g., “does the artificial limb feel like my hand?”) and implicit (e.g., “do I react with the artificial limb as I would with my own hand?”) (de Vignemont, 2018, 2011). Indeed, explicit and implicit measures used for studying artificial limb embodiment (via the prominent rubber hand illusion paradigm) often produce conflicting results (Holle et al., 2011; Holmes et al., 2012; Rohde et al., 2011), potentially due to the involvement of meta-cognitive processes, such as suggestibility (Lush, 2020; Lush et al., 2020; Marotta et al., 2016). Therefore, one may legitimately question to what extent the term “embodiment” refers to a phenomenon of real-world relevance. There is currently a growing need for novel measures of artificial limb embodiment.

In the present study, we focus on the significant role that our hands play in a core aspect of human life—communication. Specifically, hand gestures have been shown to play an important role in how we communicate. Observed across world languages and cultures, hand gestures are a universal component of communication (Feyersein and De Lannoy, 1991). For example, co-speech gesture has been documented in congenitally blind individuals who have had no gesturing model to copy or learn from (Iverson and Goldin-Meadow, 1998). Gesturing while speaking has been shown to increase listeners' comprehension of speech, as well as convey information that is not expressed in words (Goldin-Meadow, 2003; Goldin-Meadow and Alibali, 2013). Considering that co-speech gestures are spontaneously produced by our arms and hands, this unique behavior may therefore provide information about how individuals relate to their artificial limbs. Do prosthesis users use their prosthesis to produce gestures along with speech? If so, does this spontaneous behavior relate to their other functional prosthesis usage habits? And can increased gesture with a prosthesis be taken as a marker for increased embodiment?

**Figure 1:**
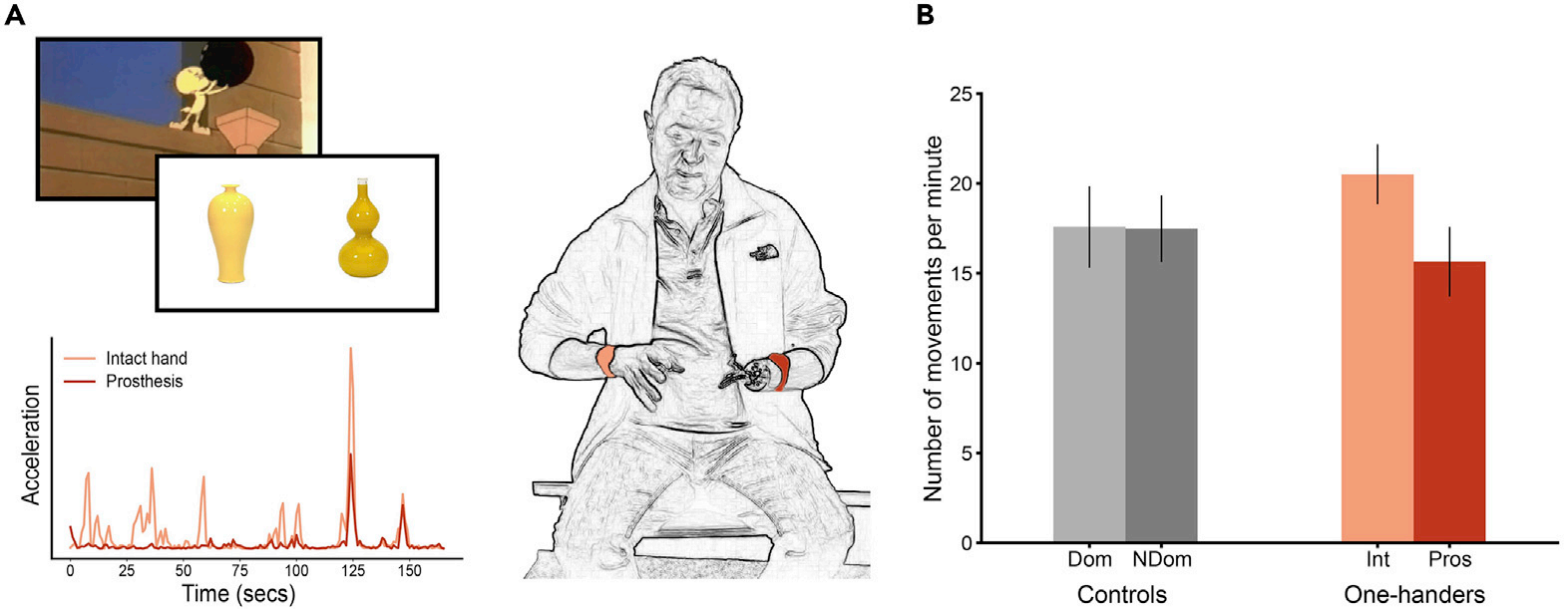
Measuring Gesticulation Behaviour (A) Experimental paradigm. Top left: example stimuli from the paired object task and a frame from an animated video shown during the storytelling task. In the paired objects task, participants were asked to describe images of object pairs that looked very similar to one another and were difficult to characterize using verbal description alone (Lu and Goldin-Meadow, 2018). In the storytelling task, participants watched two short animated video clips and described them in as much detail as possible to a (presumed to be) naive listener (McNeill, 1992; McNeill and Levy, 1982). Bottom left: pre-processed accelerometry data of a one-handed participant gesturing with their arms during the tasks. Light red indicates measured acceleration of the intact hand; dark red indicates prosthesis. Right: An illustration of a one-handed participant wearing the watch-like acceleration monitors used to measure gesticulation behavior. (B) Number of movements analysis*.* One-handers and two-handers performed the same number of gestures taking both hands into account. However, we did find an interaction (F_(1,38)_ = 4.25, p = 0.046) between arm and group: one-handers performed more movements with their intact arm than with their prosthesis (t_(24)_ = 2.94, p = 0.007), whereas two-handers produced an equal number of movements with their two arms (t_(14)_ = 0.088, p = 0.93, BF_10_ = 0.263). Bars depict group mean; error bars represent standard error of the mean (SEM). Dom, dominant arm; NDom, nondominant arm; In, intact arm; Pros, prosthetic limb.

Using accelerometry, we aimed to characterize communicative gestures performed by one-handed individuals with congenital and acquired unilateral upper limb loss (hereafter one-handers) who use an upper-limb prosthesis. By characterizing this gesturing behavior, we sought to investigate how prosthesis gesturing relates to both prosthesis use in everyday life and perceived prosthesis embodiment. One-handers engaged in two tasks designed to probe co-speech gesture behavior (gesticulation; Figure 1A) while being naive to the purpose of the study. Daily prosthesis use and perceived sense of embodiment of the prosthetic limb were measured using questionnaires. We hypothesized that better prosthesis use in daily activities will relate to increased prosthesis embodiment and more prosthesis gesture.

## Results

### Prosthesis Functional Daily Use and Perceived Embodiment Are Closely Linked

**Figure 2:**
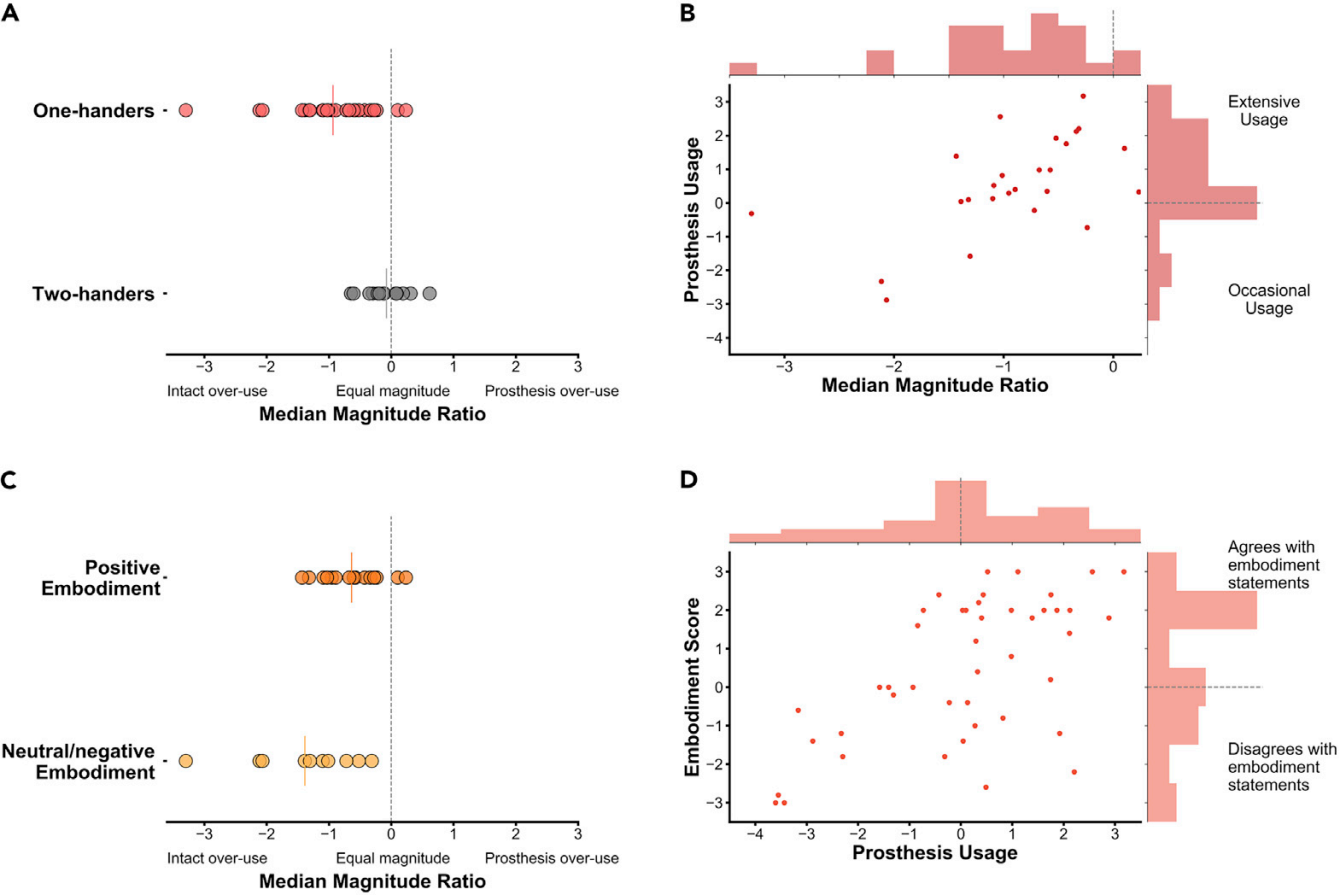
Gesticulation Behavior, Prosthesis Use, and Embodiment (A–C) The median magnitude ratio (MMR) reflects how much each arm contributes to the overall size of gesture movements performed during the task. (A) MMR values across groups; two-handers performed relatively equal size arm movements when gesturing, whereas one-handers were significantly lateralized toward their intact arm (U = 47, p < 0.001). (B) Increased daily prosthesis use (measured by questionnaires) associated with increased incorporation of the prosthesis into gestures (measured by MMR) (rho_(23)_ = 0.55, p = 0.005). (C and D) Embodiment scores reflect individuals' mean response to five subjective embodiment statements. (C) MMR values across individuals who responded positively versus neutral/negatively to prosthesis embodiment statements (for example, “it seems like the prosthesis is my hand,” “it seems like the prosthesis is part of my body”). Individuals who positively embody their prosthesis show increased incorporation of their prosthesis into gestures (Mann-Whitney U = 35, p = 0.03). (D) Greater prosthesis use is associated with greater perceived prosthesis embodiment (rho_(42)_ = 0.53, p < 0.001). In (A and C) solid colored lines indicate the group mean MMR. In (B and D) the dashed lines in the histograms indicate the position of zero. See also Figure S2.

We first examined the relationship between daily prosthesis use and perceived prosthesis embodiment in our larger cohort of one-handed individuals (n = 44; Tables 1 and S1). We found a significant correlation between these two measures (rho_(42)_ = 0.53, p < 0.001), revealing that prosthesis use and prosthesis embodiment are closely linked (Figure 2D). Both wear time and functional use (the two components comprising our daily use score) were found to correlate with perceived prosthesis embodiment (rho_(42)_ = 0.46, p = 0.002, rho_(42)_ = 0.48, p < 0.001, respectively). However, self-report questionnaire-based measures of embodiment are arguably crude and prone to bias and inter-individual differences (e.g., in suggestibility; Lush et al., 2020). We therefore turned to a more implicit measure of embodiment—spontaneously produced communicative hand gestures with the prosthesis.

**Table 1:** Demographic Information on Participants

	One-Handers—Full Cohort (N = 44)	One-Handers—Subset in the Gesticulation Accelerometry Study (N = 25)	Two-Handers (N = 15)
Age (years) (mean ± SD)	47.32 ± 11.98	46.96 ± 11.77	44.53 ± 14.36
Cause of limb loss Years since amp (mean ± SD)	23 Congenital limb deficiency	15 Congenital limb deficiency	NA
	21 Amputation in adulthood 17.33 ± 11.91 years ago	10 Amputation in adulthood 17.1 ± 12.65 years ago	
Gender	29 M	15 M	10 M
	15 F	10 F	5 F
Missing hand/Nondominant side	29 L	16 L	10 L
	15 R	9 R	5 R
Prosthesis type^a^	14 Cos	9 Cos	NA
	13 Mech	3 Mech	
	15 Myo	13 Myo	
Prosthesis wear time weekly hours (mean ± SD)	65.83 ± 35.09 Range: 0–126	72.82 ± 29.83 Range: 6–112	NA
PAL score (mean ± SD)	0.43 ± 0.23 Range: 0–0.89	0.49 ± 0.21 Range: 0.07–0.89	NA
Embodiment score (mean ± SD)	0.47 ± 0.1.84 Range: -3–3	0.75 ± 1.68 Range: −2.2–3	NA

Gender: M = male, F = female. Missing hand in one-handers and nondominant hand in two-handers: R = right hand, L = left hand; Amp level = level of limb loss: Pros type = prosthesis type worn for the greatest time in a typical week: Cos = cosmetic, Mech = mechanical, Myo = myo-electric. Pros wear time = hours per week during which a prosthesis was typically worn. PAL score = functional ability with prosthesis as determined by PAL questionnaire: 0 = minimum function, 1 = maximum function. See also Table S1.

a Prosthesis type is not reported for 2 individuals in the full cohort, who had a prosthesis they could wear but did not use at all.

### One-Handers Perform More and Larger Movements with Their Intact Arm Than with Their Prosthesis while Gesturing

Looking first at overall gesture behavior, we found that one-handers (n = 25) and two-handed controls (n = 15) did not differ in the overall number of movements per minute (as measured by accelerometry) that they produced with their two arms taken together during the gesture tasks (rmANOVA group effect: F_(1,38)_ = 0.045 p = 0.83; Figure 1B). However, we did find an interaction between arm and group (F_(1,38)_ = 4.25, p = 0.046): two-handers produced an equal number of movements with their two arms (t_(14)_ = 0.088, p = 0.93, BF_10_ = 0.263); in contrast, one-handers produced more movements with their intact arm than with their prosthetic arm (t_(24)_ = 2.94, p = 0.007).

We next calculated the median magnitude ratio (MMR) (Bailey et al., 2015; Chadwell et al., 2016). The MMR is a relative (laterality) measure that reflects how much each arm contributed to the overall size of gesture movements on a second-by-second basis. The MMR is a better validated measure than number of movements because it does not depend on an arbitrary threshold to separate movements. As the MMR is a more sensitive measure than number of gestures, it will be used in all subsequent analysis (see Figure S2, which report two alternative gesture measures with similar results). We found that one-handers made bigger movements with their intact arm than with their prosthesis arm (negative MMR) relative to controls, who showed movements of equal magnitude across the two arms (U = 47, p < 0.001; Figure 2A).

The laterality of gesture movement magnitude (MMR) correlated significantly with a laterality measure extracted from offline video coding (rho_(18)_ = 0.76, p < 0.001; Figure S1A; see Transparent Methods and Figure S1B for further validation). Thus, although one-handers gestured just as much as two-handed controls, the distribution of their gestures across hands, and the character of these gestures, differed: one-handers performed more and larger movements with their intact arm than with their prosthesis arm; two-handers performed relatively symmetrical movements with their two arms.

### Gesture Behavior Does Not Depend on Users' Developmental Period of Hand Loss or Prosthesis Type

Our one-handed sample consists of two sub-groups: individuals with congenital limb loss and amputees, who are known to adopt different adaptive strategies to compensate for their missing limb (Makin et al., 2013). We therefore determined whether cause of limb loss has an effect on prosthesis use during gesture (see Figure S3B for a comprehensive account of the relationship between cause of limb loss and embodiment). No significant effect was found when directly comparing the laterality in magnitude (MMR) or amount (numbers of movements) across the two arms of the two sub-groups (MMR: U = 59, p = 0.4; numbers of movements: U = 66, p = 0.64).

Another way in which one-handers differ is the type of prosthesis they use. Nine of our participants used a cosmetic prosthesis, a passive hand-shaped apparatus; 16 used an active prosthesis, either a mechanical hook (n = 3) or myoelectric prosthesis (n = 13). Again, no significant effect was found when directly comparing the laterality value of users with passive versus active prostheses (MMR: U = 71, p = 0.98; number of movements: U = 69, p = 0.89). The type of prosthesis thus does not affect how it will be incorporated into co-speech gesture.

### Prosthesis Gesture Reflects Daily Prosthesis Functional Use

We next examined the relationship between use of the prosthesis for gesturing and use of the prosthesis for daily activities. We found that prosthesis users who incorporated their prosthesis into their gestures in greater magnitude (resulting in a higher MMR) also tended to have a higher prosthesis use score, based on questionnaires probing functionality and wear frequency (rho_(23)_ = 0.55, p = 0.005; Figure 2B). This effect is robust and remains significant when controlling for cause of limb loss and prosthesis type. Greater prosthesis use during daily life is thus associated with greater use of the prosthesis while gesturing.

We next examined this link between prosthesis use in gesturing and use in daily life in relation to cause of amputation (congenital versus acquired) and prosthesis type (passive versus active) using parametric statistics. To meet the requisite statistical assumptions, we removed an outlier on the laterality measure (participant code: “aa11”) from the analysis. An ANCOVA with sub-group and prosthesis type as fixed effects, and daily prosthesis use score as a covariate, revealed no significant sub-group effect on the laterality measure during gesturing (cause of limb loss: F_(1,20)_ = 0.8, p = 0.38; prosthesis type: F_(1,20)_<0.01, p = 0.99). Neither cause of limb loss nor type of prosthesis thus appears to play a key role in determining gesticulation behavior with the prosthesis. Importantly, the relationship between prosthesis gesturing (as captured in the MMR) and daily prosthesis use remained significant in this analysis (F_(1,20)_ = 13.97, p = 0.001), which highlights the robustness of the relationship between use of the prosthesis for gesturing and use of the prosthesis for daily activities. This additional analysis further confirms that one-handers' gesture behavior is strongly related to their level of prosthesis use in daily activities and not to the cause of amputation or the type of prosthesis used.

### Positive Perceived Prosthesis Embodiment Associates with Increased Prosthesis Use in Gestures

We next examined the relationship between gesture movements and prosthesis embodiment. Individuals varied in their responses to the subjective embodiment statements, producing a range from −3 (strongly disagree) to 3 (strongly agree) where 0 is a neutral response. As a whole, one-handed participants tended to marginally, although significantly, report that they experienced embodiment of their prosthesis (mean = +0.75, difference from zero: t_(24)_ = 2.23, p = 0.035). We found similar effects when we looked at the full study cohort (i.e., all of the participants including those whose data were not included in the accelerometry part of the study); the full cohort showed a trend toward positive embodiment (n = 44, mean = 0.47; difference from zero t_(43)_ = 1.685, p = 0.099). Our embodiment questionnaire included a control question, “it seems like I have three hands,” which received an averaged rating of −2.89 in our full cohort. In other words, participants strongly disagreed with this statement, indicating that the overall neutral responses to the embodiment questions did not result from lack of engagement with the statements.

We next divided the one-handed participants into two groups based on whether they reported positive (score >0) or neutral/negative (score ≤0) embodiment of their prosthesis. We then looked at the gesture laterality profiles of these sub-groups and found that one-handers who reported positive embodiment (n = 15) used their prosthesis more when gesturing (i.e., higher MMR) than one-handers who reported neutral/negative embodiment (U_(23)_ = 35, p = 0.03; Figure 2C). When analyzing the full range of embodiment scores, we found a trend toward a positive relationship between embodiment score and the laterality of gesture magnitude (MMR) (rho_(23)_ = 0.37, p = 0.07; Figure S3): the more positively one-handers respond to embodiment statements regarding their prosthesis, the more symmetrical their gestures are. Subjective reports of perceived embodiment are thus only marginally associated with spontaneous gesture. Therefore, it appears that the implicit measure of gesture laterality better captures day-to-day prosthesis use.

## Discussion

Here, we demonstrate that artificial limbs are regularly used to produce co-speech gestures, offering further demonstration of the ubiquity of gesture production in human communication. Despite hand loss (either congenitally or through amputation later in life), and independent of prosthesis type, one-handers gesture just as much as two-handers do. However, the profiles of the gestures produced by one- versus two-handers differed. Specifically, one-handers produced lateralized gestures, favoring their intact hand in number and magnitude, whereas two-handers produced symmetrical gestures that were equally dispersed across both hands. Furthermore, one-handers who gesture more with their prosthesis, and produced more symmetrical gesture patterns, were also shown to report more positive feelings of prosthesis embodiment and greater prosthesis use in everyday life. As gesticulation was spontaneously generated by the participants, and was never explicitly mentioned as part of the task, it is resistant to recent criticisms relating to inherent biases in the induction of embodiment measures, such as the rubber hand illusion (Lush, 2020; Lush et al., 2020). Our findings thus provide a novel means for implicitly indexing embodiment in artificial limbs, with relevance for how artificial limbs are being operated in real-world contexts.

To our knowledge, our findings are the first empirical demonstration of a strong relationship between reported prosthesis embodiment and everyday prosthesis use (although see Graczyk et al., 2018 for results from two individuals). Despite technological progress, individuals with congenital and acquired missing upper limbs continue to report low functionality and use of their prostheses, and instead prefer to over-rely on their intact hand (Jang et al., 2011). In as many as 40% of cases, one-handers abandon their prostheses altogether (Raichle et al., 2008). Prosthesis abandonment often occurs after being fitted with a customized prosthesis (Østlie et al., 2012), resulting in wasted resources. Successful prosthesis use is difficult to predict, and often can only be determined by trial and error over the course of months. A further challenge is in quantifying how one-handers use their prostheses in day-to-day life (Chadwell et al., 2020). Past attempts to develop an objective prosthesis use measure have used activity monitors worn across several days. However, at present, these studies are limited by the type of information that can be extracted about the specific activities that the participant was performing. At one extreme, activity can be recorded from participants swinging their limbs while walking, without making overt use of the prosthesis. At another extreme, using the prosthesis to hold the wheel while driving will not be recorded as vigorous activity, although it is a focused use of the prosthesis and critical in terms of daily activities. Although this technology is promising, it needs to be developed further to support clinical purposes. Until such time, questionnaires provide us with a proxy measure to prosthesis usage in daily life.

Previous studies have focused primarily on prosthesis dexterity (e.g., grasping and manipulating objects), which is arguably not synonymous with prosthesis adoption. Here we outline a radically different hallmark of prosthesis use––how do you spontaneously use your hands to convey meaning when talking? We show that prosthesis gesticulation relates to both how functional the prosthesis is in daily life and how it is experienced in terms of embodiment. Nevertheless, it is important to highlight that our findings are correlational, and thus it is impossible to infer whether increased embodiment causally contributes to enhanced prosthesis use, as extensively speculated before. With this important caveat in mind, we propose that incorporating a prosthetic limb into gesturing reflects a natural yet easily quantifiable level of immersion of the prosthesis into the user's body and behavior–– an ultimate goal of any human-machine interface. As gesticulation requires minimal skill and is independent of the device's function, it could hypothetically be used to inform engineering design of prostheses and other wearable technologies.

Finally, we propose that accelerometry-based gesticulation analysis of one-handers could be used as a simple and objective clinical measure of prosthesis embodiment and everyday use. Assessing gesticulation is quick (up to 10 min), requires no training, can be used across prosthesis types, and provides a quantitative and objective measure (see Resource Availability below for open-source analysis codes). Importantly, gesticulation can be measured implicitly and objectively, thus minimizing user and clinician biases (Pareés et al., 2012). As such, gesticulation may provide an ideal point-of-care clinical assessment for tracking the efficacy of upper-limb rehabilitation over time.

### Limitations of the Study

Although gesticulation holds potential for predicting successful prosthesis use and embodiment, a key limitation of the current study is the lack of longitudinal measures. This limits our ability to make causal inferences about the relationship between prosthesis gesture and functional use. This caveat also prevents us from demonstrating the potential predictive power of gesticulation in longer-term prosthesis use. Further research should determine whether gesticulation during fitting/early training can predict prosthesis adoption, which is a key issue in prosthesis rehabilitation.

In this study we have argued for a more objective measure for prosthesis embodiment and use, emphasizing that self-report can be misleading. However, to interpret our objective measure we have used two self-report questionnaires. Although both measures have been validated (see Methods), it is true that they are still susceptible to suggestibility and other biases. As questionnaires are the most commonly used form of evaluation in this field, it is necessary to align our present findings with standard procedures and previous literature. We hope future studies will be able to use our objective measure without having to rely on questionnaires and to even go a step further and relate our measure to other objective measures of prosthesis use, such as use measures extracted from activity monitors worn in daily life.

### Resource Availability

#### Lead Contact

Further information and requests for resources should be directed to and will be fulfilled by the Lead Contact, Tamar R. Makin (t.makin@ucl.ac.uk).

#### Materials Availability

All stimuli used in the described tasks can be found at the Open Science Framework repository: https://osf.io/spt2a/.

#### Data and Code Availability

Analysis code along with data used to generate the figures can be found at the Open Science Framework repository: https://osf.io/spt2a/.

## Methods

All methods can be found in the accompanying Transparent Methods supplemental file.

## Supplemental Information

Document S1. Transparent Methods, Figures S1–S3, and Table S1
