# Supplementary material for: Talking with Your (Artificial) Hands: Communicative Hand Gestures as an Implicit Measure of Embodiment
Source: iScience. 2020 Oct 6;23(11):101650. doi: 10.1016/j.isci.2020.101650 (PMC7578755; doi:10.1016/j.isci.2020.101650)
Supplement: Document S1. Transparent Methods, Figures S1–S3, and Table S1 [file mmc1.pdf]

iScience, Volume *23*

Supplemental Information

Talking with Your (Artificial)

Hands: Communicative Hand Gestures

as an Implicit Measure of Embodiment

Roni O. Maimon-Mor, Emeka Obasi, Jenny Lu, Nour Odeh, Stephen Kirker, Mairéad MacSweeney, Susan Goldin-Meadow, and Tamar R. Makin

Supplementary Figures

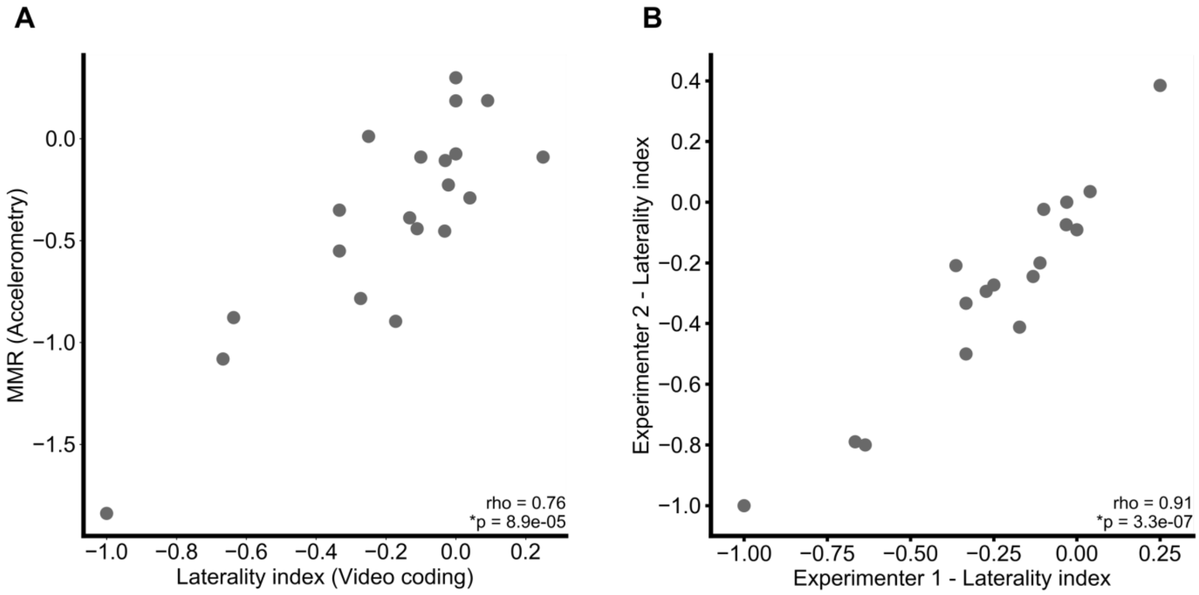

Figure S1. *Measurement validation using Offline video coding*. (A) Correlation between the laterality index calculated from offline video coding, on a subset of participants, and the MMR ($rho_{(18)} = 0.76$, $p < 0.001$). (B) Test-retest reliability of the offline video coding method. The laterality measure was validated by an additional experimenter. The measurement was found to be stable across the two separate experimenters ($rho_{(16)} = 0.96$, $p < 0.001$).

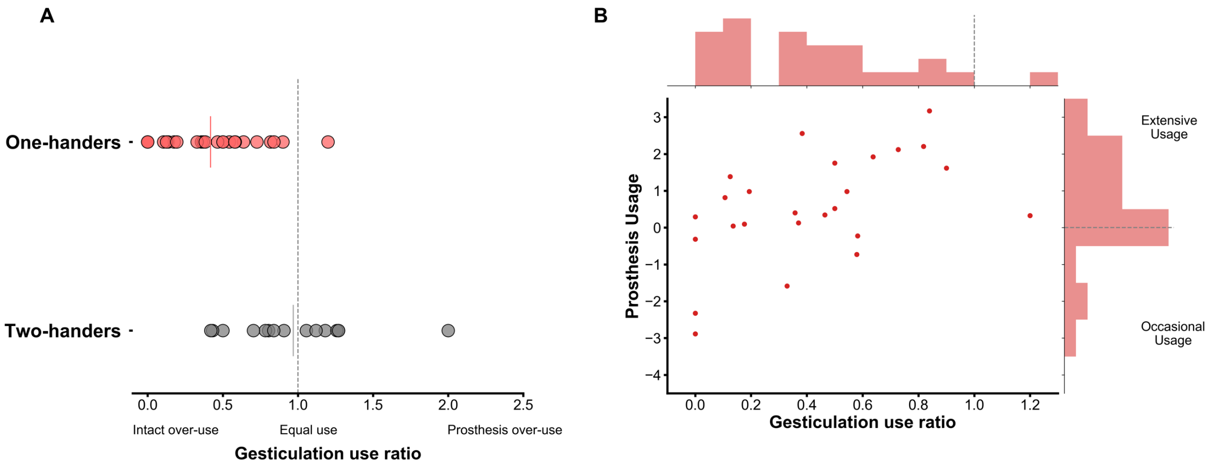

Figure S2. *Results of analysis with alternative gesture measure: Use ratio.* Related to Figure 2. The use ratio quantifies the total duration of one arm's movement with respect to the other (Lang et al., 2017). Unlike the MMR, use ratio is sensitive to the presence of movement in each second but not to the magnitude of the movement A value between 0 and 1 indicates

greater use of the intact/dominant arm than the prosthetic/non-dominant arm; a value of 1 indicates equal use between both arms; and a value larger than 1 indicates greater use of the prosthetic/non-dominant arm than the intact/dominant arm. Using this measure in the analysis produced similar results to those reported in the main text. (A) Use ratio across groups; two-handers performed movements with both hands equally when gesturing, while one-handers were significantly lateralised towards their intact hand ($U_{(38)}$ = 54, p < 0.001). Solid coloured vertical lines indicate the group mean (B) Increased daily usage associated with increased incorporation of the prosthesis into gestures as measured by the use ratio ($rho_{(23)}$ = 0.55, p = 0.004).

To demonstrate that our results are not specific to complex measures we also calculated the standard deviation of the acceleration time-series for each arm. Using this measure in the analysis produced similar results to those reported in the main text. Repeating the same statistical analysis performed on the number of movement measure, the repeated measure ANOVA showed the same significant hand*group interaction F(41)=9.310, p=0.004, with one-handers showing more variance in their intact hand compared to their prosthesis. Using the log ratio of the standard-deviation of the two arms, we also found a significant correlation between daily usage is associated and prosthesis use in gestures (r(23)=0.54, p=0.005).

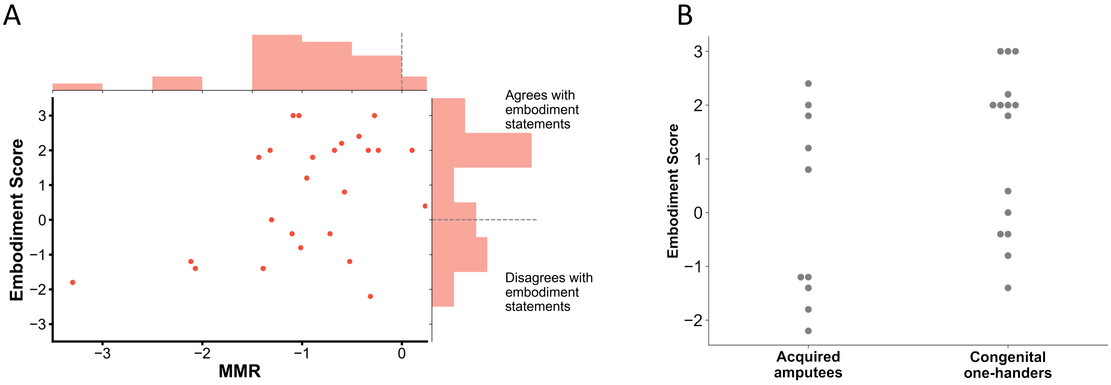

Figure S3. *Embodiment supplementary results. (A) Embodiment and gesture behaviour*. Greater perceived embodiment was loosely associated with increased incorporation of the prosthesis into gestures as measured by MMR ($rho_{(23)}$ = 0.37, p = 0.07). (B) *Embodiment and cause of limb-loss***.** Across the full cohort (n=44) we found no differences in embodiment scores between individuals with acquired and congenital limb-loss (Mann-Whitney U = 175.5, p = 0.12). Within our gesture subset (n=25, plotted in panel B) there was a trend toward significance in the same comparison (Mann-Whitney U = 42, p = 0.07). In the main analysis examining the relationship between embodiment and gesture behaviour (as measured by MMR) we have split the one-handed group to participants who reported

positive and neutral/negative embodiment. There was no significant difference between individuals with acquired and congenital limb-loss in their association to the positive or neutral/negative embodiment groups (Chi-square = 0.69, $p = 0.40$).

Participant	Age	Y Since Amp	Gender	Amp Side	Amp level	Amp cause	Prosthesis Type	PLS	PLP	SP	Pros wear time	PAL	US	EM
AA01	58	14	M	L	TR	Trauma	Myo	15	0	0	119	0.5	1.87	2
AA02	46	16	F	L	TR	Trauma	Myo	25	8	0	56	0.59	0.49	-2.6
AA03	50	3	F	L	TR	Trauma	Mech	0	0	0	77	0.44	0.44	2.4
AA04	53	34	M	L	TH	Trauma	Mech	90	25	20	48	0.2	-1.40	0
AA05	21	1	M	R	TR	Trauma	None	50	30	17.5	0	0.04	-3.44	-3
AA06*	42	18	M	R	TR	Trauma	Cos	13.33	16	90	35	0.07	-2.33	-1.2
AA07*	61	21	M	L	TR	Trauma	Cos	95	50	0	105	0.67	2.21	-2.2
AA08*	60	42	M	R	TR	Trauma	Mech	100	60	5	87.5	0.28	0.05	-1.4
AA09	65	37	M	R	TH	Trauma	Mech	90	0	12	98	0.46	1.11	3
AA10	47	21	M	R	TH	Trauma	Cos	60	8	0	84	0.3	0.03	2
AA11*	68	12	M	L	TR	Trauma	Mech	0	0	7.5	35	0.54	-0.31	-1.8
AA12*	49	5	M	R	TR	Vascular disease	Cos	14	0	10	42	0.59	0.10	2
AA13	57	29	M	L	TR	Trauma	Mech	6.25	0	17.5	65	0.11	-1.31	-0.2
AA14*	53	33	M	L	TR	Trauma	Myo	0	0	0	98	0.43	0.98	0.8
AA15	28	10	F	R	TR	Trauma	Mech	85	21.67	0	2	0	-3.55	-2.8
AA16	29	11	M	L	TR	Trauma	None	100	16	16	0	0	-3.61	-3
AA17*	43	20	M	R	TR	Trauma	Myo	37.5	0	10	98	0.61	1.75	2.4
AA18*	55	12	M	L	TR	Trauma	Myo	0	0	0	98	0.65	1.92	-1.2
AA19	61	17	M	L	TR	Trauma	Mech	30	17.5	20	91	0.74	2.11	1.4
AA21*	30	3	M	L	TR	Trauma	Myo	20	18	60	49	0.59	0.29	1.2
AA22*	46	5	M	R	TR	Trauma	Myo	70	2.5	16	56	0.57	0.40	1.8
AC01	51		F	L	TR	Congenital	Cos				7	0.26	-2.30	-1.8
AC02	47		M	L	TR	Congenital	Mech				84	0.7	1.75	0.2
AC03*	45		F	L	TR	Congenital	Myo				63	0.46	0.13	-0.4
AC04*	26		M	L	TR	Congenital	Mech				6	0.13	-2.88	-1.4
AC05*	55		F	L	TR	Congenital	Cos				112	0.3	0.82	-0.8
AC06*	63		M	L	TR	Congenital	Cos				87.5	0.35	0.35	2.2
AC07	35		M	L	TR	Congenital	Cos				56	0.28	-0.84	1.6
AC08*	26		F	L	TR	Congenital	Cos				84	0.24	-0.22	-0.4
AC09*	49		M	L	TR	Congenital	Myo				91	0.57	1.39	1.8
AC10	42		M	L	TR	Congenital	Cos				56	0.54	0.28	-1
AC11	66		F	R	TR	Congenital	Cos				42	0.35	-0.93	0
AC12*	56		F	R	TR	Congenital	Cos				98	0.43	0.98	2
AC13	53		M	L	TH	Congenital	Mech				63	0.33	-0.43	2.4
AC14	42		M	L	TR	Congenital	Mech				2	0.09	-3.17	-0.6
AC15*	55		F	L	TR	Congenital	Myo				105	0.65	2.12	2
AC16*	38		F	R	TR	Congenital	Cos				84	0.67	1.62	2
AC17*	29		M	L	TR	Congenital	Myo				70	0.46	0.33	0.4
AC18*	53		F	L	TR	Congenital	Cos				48	0.65	0.52	3
AC20*	52		F	R	TR	Congenital	Myo				32.5	0.26	-1.58	0
AC21*	32		F	R	TR	Congenital	Myo				40	0.41	-0.73	2
AC22	57		M	R	TR	Congenital	Mech				126	0.69	2.88	1.8
AC23*	47		F	L	TR	Congenital	Myo				84	0.89	2.56	3
AC25*	41		M	L	TR	Congenital	Myo				112	0.85	3.17	3
CO01*	48		M											
CO04*	59		M											

CO05*	27		F											
CO07*	35		M											
CO08*	34		F											
CO10*	70		M											
CO12*	18		F											
CO13*	67		M											
CO14*	50		M											
CO15*	51		F											
CO16*	36		F											
CO17*	41		M											
CO18*	33		M											
CO19*	45		M											
CO21*	54		M											

Table S1. *Demographic details of all participants.* Related to Table 1. Participant: AA = acquired amputee, AC = congenital one-hander, CO = two-handed control; participants marked with an asterisk were included in the gesticulation task. Y since amp = years since amputation. Gender: M = male, F = female. Amp Side = side of limb loss or non-dominant side: L = left, R = right. Amp level = level of limb loss: TR = trans-radial, TH = trans-humeral. Pros type = preferred type of prosthesis: Cos = cosmetic, Mech = mechanical, Myo = myo-electric. PLS = phantom limb sensation. PLP = phantom limb pain. SP = stump pain. Chronic PLS, PLP and SP were calculated by dividing maximum intensity of pain (0-100) by frequency (1 = all the time, 2 = daily, 3 = weekly, 4 = several times per month, and 5 = once or less per month). Pros Time = typical number of hours prosthesis worn per week. PAL = functional ability with prosthesis as determined by PAL questionnaire (0 = minimum function, 1 = maximum function). US = prosthesis usage score: +3 = maximum usage, -3 = minimum usage. EM = prosthesis embodiment score; +3 = maximum agreement with embodiment statements, -3 = maximum disagreement with embodiment statements.

Transparent Methods

Participants

44 one-handed individuals were recruited for this study: 21 unilateral acquired amputees (mean age ± std = 48.67 ± 12.9, 18 male, 12 with intact right hand), and 23 individuals with congenital unilateral upper-limb loss (age ± std = 46.09 ±11.22, 11 male, 17 with intact right hand; see Tables 1 and S1 for full demographic details). Sample size was based on recruitment capacities considering the unique populations we tested. Nineteen individuals from the full set of participants were excluded from the gesticulation-accelerometry analysis for the following reasons: Issues with data storage (n=7); trans-humeral level limb-loss (n=4); did not participate in gesture task (n=2); rated their typical weekly prosthesis use as 0 hours, an exclusion criterion of the study (n=2); aware of the purpose of the task before participating (n=1). Three participants did not produce any co-speech gestures during the tasks, and since our main measure is a relative measure between the two hands, they were not included in the analysis. Nevertheless, including them in the group comparison of number of gestures produced similar results to those reported earlier.

A total of 25 participants (10 acquired amputees and 15-congenital one-handers) were included in the gesticulation-accelerometry analysis, together with 15 age, gender, and handedness matched two-handed controls (see Table 1). All participants filled in the prosthesis-use and prosthesis embodiment questionnaires. There were no significant differences between one-handers and two-handers in age ($t_{(38)} = 0.565$, $p = 0.58$), gender (Pearson chi-square = 0.000, $p = 1$), and handedness during the study (intact hand in one-handers and dominant hand in controls; Pearson chi-square = 0.03, $p = 0.86$). We note that, for acquired amputees, we consider functional handedness and refer to their intact hand as their dominant hand, regardless of their pre-amputation practices. The study's sample size of amputees prevents us from exploring effects of losing a dominant vs. non-dominant hand. Participants were recruited to the study between October 2017 and December 2018, based on the guidelines in our ethical approval UCL (REC: 9937/001) and in accordance with the declaration of Helsinki. The following inclusion criteria were taken into consideration during recruitment: (1) 18 to 70 years old, (2) MRI safe (for the purpose of other tasks conducted in the scanner), (3) no previous history of mental disorders, (4) for one-handers, owned at least one type of prosthesis during recruitment, (5) for acquired amputees, amputation occurred at least 6 months before recruitment. All participants gave full written informed consent for their participation, data storage, and filming.

Tasks

Participants engaged in two tasks in which they were presented with a series of short video clips and images designed to probe gesticulation. The first was a storytelling task, which is a well-established gesture elicitation task (McNeill, 1992; McNeill and Levy, 1982) in which gestures are spontaneously produced during narrative discourse. Participants were shown two video clips of the cartoon 'Tweety and Sylvester' (see Figure 1A). After each clip, a listener, who the participants were told was naïve to the videos, entered the room and sat opposite the participant. Each participant was then required to recall and describe the videos back to the listener in as much detail as possible.

The second task was the Paired Objects task. In each of the 4 trials, participants were presented with images of two items and asked to describe them in as much detail as possible to the listener. Each image displayed a pair of similar looking objects, specifically chosen to be difficult to describe using words alone and therefore optimal for eliciting gestures (see Figure 1A). This method was developed by Lu & Goldin-Meadow in a study that focused on the depiction of object shape and size in handshapes in deaf signers (Lu and Goldin-Meadow, 2018). In addition to the listener present in the room, the participants were told that an additional person would watch the video of their descriptions and should be able to recognise the images based on the description. This instruction was added to emphasize the need for a thorough description. The listener was included as previous research suggests that individuals gesture more when there is a visible listener, compared to no listener or a listener hidden behind a screen (Alibali et al., 2001). The stimuli were displayed on a computer screen using a Microsoft PowerPoint presentation, each pair was on a separate slide. When the participants indicated that they had finished describing the current pair, the experimenter pressed a button to move to the next trial.

In both parts, participants were naïve to the purpose of the task since being aware that the task was designed to elicit gesture could have interfered with their performance. Participants were seated to face the camera, which recorded the task.

Gesture measurements

To capture gesture behaviour, GENEActiv accelerometers (ActivinsightsLtd, Kimbolton, Cambridgeshire, UK) and AX3 accelerometers (Axivity, Newcastle upon Tyne, UK) were used. An accelerometer was placed on each of the participant's arms, on both wrists for control participants, and on the intact wrist and 'prosthesis wrist' for one-handed participants. The participants were not informed of the function of the accelerometers prior to the task to minimise any effect it might have had on performance. The accelerometers were set to record

tri-axial data with a sampling frequency of 100Hz and range of ±8g, as well as the time stamp for each recorded signal. Raw acceleration data was extracted and pre-processed using MATLAB (version R2017a; Mathworks, Natick, MA, USA). The data from the left and right upper-limb/prosthesis were first synchronised with each other using the recorded time stamps to account for any minor sampling frequency errors between each device. The data were then plotted and visually inspected for any anomalous recordings, and the plot of the data was synchronised with the video clip of the task to ensure that the correct portions of data were analysed.

Movements along the 3 axes were combined [$\sqrt{x^2+y^2+z^2}$] and bandpass filtered using a 4th order Butterworth filter between the frequencies 0.2Hz and 15Hz to remove high frequency noise and gravitational artefact. Bandpass frequencies were chosen based on previous accelerometry studies looking at upper limb activity (Mannini et al., 2013; Schaefer et al., 2014; van Hees et al., 2011).

The filtered data was used to quantify gesture movements in two separate ways: (1) The total number of movements performed with each arm was calculated using a sliding window method, whereby an individual gesture was defined as each 400ms window of data in which there was movement (defined as an acceleration value ≥0.2g) that was preceded and succeeded by a window of no movement (<0.2g) (Makin et al., 2013). We note that our results do not depend on the arbitrary choice of time-window, as similar results were found with a 200ms and a 600ms window. To account for differences in recording times between participants, the total number of movements performed per minute of talk was calculated. (2) The median magnitude ratio (MMR) of the accelerometry data was calculated to investigate how much each arm contributed to the overall size of gesture movements performed during the task (Lang et al., 2017) . This method has been previously used successfully to quantify every-day behaviour in impaired individuals and specifically amputees (Bailey et al., 2015; Chadwell et al., 2016). The data were then down-sampled to 1Hz. The magnitude ratio (MR) between the intact arm and prosthesis was calculated for each second as [$MR = ln\frac{Prosthesis\ counts}{Intact\ counts}$]. A value of 0 indicating equal movement of both arms, <0 indicating greater size movements with the intact/dominant arm relative to the prosthetic/non-dominant arm, and >0 indicating greater size movements with the prosthetic/non-dominant arm relative to the intact/dominant arm. To demonstrate that our results are not specific to a measure based on magnitude, the analyses were repeated using a similar measure that is insensitive to magnitude (see Supplementary Figure S2).

Six participants (4 one-handers and 2 controls) produced co-speech gestures in only one of the two tasks. For these participants, only data from the task during which they gestured was analysed; data from both tasks were analysed together for the remainder of the participants.

Gesture measurements validation

To validate the accelerometry data, the movement laterality for a subset of the participants (n=20) was also calculated using offline video-coding of the Paired Objects task. Using the ELAN software, (ELAN v5.7, The Language Archive, Nijmegen, The Netherlands) (Lausberg and Slöetjes, 2008), separate gestures were manually coded and labelled based on their laterality. Gestures were labelled as follows: involving the dominant/intact hand only; involving the non-dominant/prosthesis hand only; or involving both hands/hand+prosthesis. The end of a gesture was identified based on a change in hand position, a change in verbal content, or by a return to resting position of the hands. For each participant and for each task, the percentage of gestures for each laterality label was calculated. A laterality index was then calculated as:

$$laterality = \frac{(Prosthesis + Both) - (Intact + Both)}{Intact + 2 * Both + Prosthesis} = \frac{(Gestures\ Involving\ Prosthesis) - (Gestures\ Involving\ Intact)}{All\ Gestures}$$

Giving a value between -1 and +1, with 1 indicating total lateralisation towards the prosthetic/non-dominant hand, 0 indicating equal movement of both hands, and -1 indicating total lateralisation towards the intact/dominant hand. Coding reliability was assessed by having an additional experimenter analyse a subset of 20 participants, and compare the results between the two experimenters (see Supplementary figure S1B).

Prosthesis Use Assessment

Participants completed a questionnaire to assess the frequency and functionality of prosthesis use, which were combined to create an overall prosthesis use score (as previously used in (Maimon-Mor and Makin, 2020; van den Heiligenberg et al., 2017; Van Den Heiligenberg et al., 2018)). To determine frequency of use, participants were asked to indicate the typical number of hours per day, and days per week, that they wear their prosthesis. These scores were then used to determine the typical number of hours per week that the prosthesis was worn. To determine functionality of prosthesis use, participants were asked to complete the prosthesis activity log (PAL) (Makin et al., 2013), a modified version of the Motor Activity Log (MAL) questionnaire, which is commonly used to assess arm functionality in those with upper-

limb impairments (Uswatte et al., 2006). The PAL consists of a list of 27 daily activities (see https://osf.io/jfme8/); participants must rate how often they incorporate their prosthesis to complete each activity on a scale of “never” (0 points), “sometimes” (1 point) or “very often” (2 points). The PAL score is then calculated as the participant’s score divided by the maximum possible score, generating a value between 0 (no functionality) and 1 (maximum functionality). Prosthesis wear time and PAL were standardised using a Z-transform and summed to create a use score that included wear time and incorporation of the prosthesis in activities of daily living. The two measurements (wear time and PAL) were highly correlated (Spearman’s rho=0.61, p=0.00001).

To validate the prosthesis usage questionnaire score, 21 participants completed the prosthesis use questionnaire twice, with 1-2 years between each measurement. Since the combined usage score is a sum of z-score transformation based on the specific dataset, we calculate the reliability of PAL and wear-time frequency separately. The PAL score was found to have excellent reliability with an ICC value of .81 (two- way random-model, absolute agreement type) and 95% confidence interval of single measures = .58 –.919 [F(20,20) = 10.60 , p < .001]. For the wear time frequency, which is an ordinal 6-item non-symmetrical scale, we used Kendall’s tau-b correlation coefficient, showing a strong correlation in wear time scores ($\tau(19)=0.605$, p=0.003). These analyses confirm that both measures have good consistency, and are a reliable measure for prosthesis use.

Prosthesis Embodiment Assessment

The participants completed a 13-statement questionnaire to assess the extent of prosthesis embodiment (see https://osf.io/jfme8/). The statements were primarily adopted from a questionnaire used in rubber hand illusion studies, in which the embodiment of a rubber hand was investigated; “rubber hand” was replaced with “prosthesis” (Longo et al., 2008). Questions were divided into the following factors: Body Ownership (embodiment), Agency, Body Image, and Somatosensory. The subset of embodiment statements used here are: “*it seems like the prosthesis belongs to me”, “it seems like the prosthesis is my hand”, “it seems like the prosthesis is part of my body”, “it feels like my prosthesis is a foreign body”, “it feels like my prosthesis is fused with my body”*. We did not analyse the results from the other control questions as we did not have a strong a priori hypothesis relating to these phenomena. We will make our full data available as an open source following publication. The participants rated each of these statements on a Likert scale from -3 (strongly disagree) to +3 (strongly agree). The prosthesis embodiment score was calculated using the average score from these five statements, taking the opposite (negative) value of the ‘foreign body’ statement. A similar

embodiment questionnaire has been recently validated in a large group of lower-limb amputees (Bekrater-Bodmann, 2020).

Statistical analysis

Statistical analysis was performed using IBM SPSS Statistics for Macintosh (Version 25) and JASP (Version 0.11.1). Tests for normality were carried out using a Shapiro-Wilk test, and statistical analysis was carried out using a repeated measures ANOVA for number of movements of each arm and non-parametric tests for MMR (Mann-Whitney). All correlations were performed using two-tailed Spearman correlation. An analysis of covariance (ANCOVA) with prosthesis use as a covariate was used to test the contribution of cause of limb-loss and type of prosthesis used. We further calculated the two-way random single measures of intraclass correlations (ICCs), allowing us to assess consistency of the PAL measurement. We also used a Kendall's tau-b correlation to assess the consistency of wear-time frequency.

References

Alibali, M.W., Heath, D.C., Myers, H.J., 2001. Effects of Visibility between Speaker and Listener on Gesture Production: Some Gestures Are Meant to Be Seen. J. Mem. Lang. 44, 169–188.

Bailey, R.R., Klaesner, J.W., Lang, C.E., 2015. Quantifying Real-World Upper-Limb Activity in Nondisabled Adults and Adults With Chronic Stroke. Neurorehabil. Neural Repair 29, 969–978.

Bekrater-Bodmann, R., 2020. Perceptual correlates of successful body-prosthesis interaction in lower limb amputees: psychometric characterization and development of the Prosthesis Embodiment Scale. Sci. Rep. 10:14203.

Chadwell, A., Kenney, L., Thies, S., Galpin, A., Head, J., 2016. The reality of myoelectric prostheses: Understanding what makes these devices difficult for some users to control. Front. Neurorobot. 10.

Lang, C.E., Waddell, K.J., Klaesner, J.W., Bland, M.D., 2017. A Method for Quantifying Upper Limb Performance in Daily Life Using Accelerometers. J. Vis. Exp. 1–8.

Lausberg, H., Slöetjes, H., 2008. Gesture coding with the NGCS - ELAN system. Proc. Meas. Behav. 2008 2008, 176–177.

Longo, M.R., Schüür, F., Kammers, M.P.M., Tsakiris, M., Haggard, P., 2008. What is embodiment? A psychometric approach. Cognition 107, 978–998.

Lu, J.C., Goldin-Meadow, S., 2018. Creating Images With the Stroke of a Hand: Depiction of Size and Shape in Sign Language. Front. Psychol. 9.

Maimon-Mor, R.O., Makin, T.R., 2020. Is an artificial limb embodied as a hand? Brain decoding in prosthetic limb users. PLoS Biol. in press.

Makin, T.R., Cramer, A.O., Scholz, J., Hahamy, A., Henderson Slater, D., Tracey, I., Johansen-Berg, H., 2013. Deprivation-related and use-dependent plasticity go hand in hand. Elife 2013, 1–15.

Mannini, A., Intille, S.S., Rosenberger, M., Sabatini, A.M., Haskell, W., 2013. Activity recognition using a single accelerometer placed at the wrist or ankle. Med. Sci. Sports Exerc. 45, 2193–2203.

McNeill, D., 1992. Hand and mind: What gestures reveal about thought. The University of Chicago Press, Chicago and London.

McNeill, D., Levy, E.T., 1982. Conceptual Representations in Language Activity and Gesture, in: Jarvella, R.J., Klein, W. (Eds.), Speech, Place, and Action. Wiley, pp. 271–

295.

Schaefer, C.A., Nigg, C.R., Hill, J.O., Brink, L.A., Browning, R.C., 2014. Establishing and evaluating wrist cutpoints for the GENEActiv accelerometer in youth. Med. Sci. Sports Exerc. 46, 826–833.

Uswatte, G., Taub, E., Morris, D., Light, K., Thompson, P.A., 2006. The Motor Activity Log-28: assessing daily use of the hemiparetic arm after stroke. Neurology 67, 1189–94.

Van Den Heiligenberg, F.M.Z., Orlov, T., MacDonald, S.N., Duff, E.P., Henderson Slater, D., Beckmann, C.F., Johansen-Berg, H., Culham, J.C., Makin, T.R., 2018. Artificial limb representation in amputees. Brain 141, 1422–1433.

van den Heiligenberg, F.M.Z., Yeung, N., Brugger, P., Culham, J.C., Makin, T.R., 2017. Adaptable Categorization of Hands and Tools in Prosthesis Users. Psychol. Sci. 28, 395–398.

van Hees, V.T., Renström, F., Wright, A., Gradmark, A., Catt, M., Chen, K.Y., Löf, M., Bluck, L., Pomeroy, J., Wareham, N.J., Ekelund, U., Brage, S., Franks, P.W., 2011. Estimation of Daily Energy Expenditure in Pregnant and Non-Pregnant Women Using a Wrist-Worn Tri-Axial Accelerometer. PLoS One 6, e22922.
